# Art in an age of artificial intelligence

**DOI:** 10.3389/fpsyg.2022.1024449

**Published:** 2022-11-30

**Authors:** Anjan Chatterjee

**Affiliations:** Perelman School of Medicine, University of Pennsylvania, Philadelphia, PA, United States

**Keywords:** aesthetics, machine learning, cognition, neuroaesthetics, artist

## Abstract

Artificial intelligence (AI) will affect almost every aspect of our lives and replace many of our jobs. On one view, machines are well suited to take over automated tasks and humans would remain important to creative endeavors. In this essay, I examine this view critically and consider the possibility that AI will play a significant role in a quintessential creative activity, the appreciation and production of visual art. This possibility is likely even though attributes typically important to viewers–the agency of the artist, the uniqueness of the art and its purpose might not be relevant to AI art. Additionally, despite the fact that art at its most powerful communicates abstract ideas and nuanced emotions, I argue that AI need not understand ideas or experience emotions to produce meaningful and evocative art. AI is and will increasingly be a powerful tool for artists. The continuing development of aesthetically sensitive machines will challenge our notions of beauty, creativity, and the nature of art.

## Introduction

Artificial intelligence (AI) will permeate our lives. It will profoundly affect healthcare, education, transportation, commerce, politics, finance, security, and warfare (Ford, 2021; Lee and Qiufan, 2021). It will also replace many human jobs. On one view, AI is particularly suited to take over routine tasks. If this view is correct, then humans involvement will remain relevant, if not essential, for creative endeavors. In this essay, I examine the potential role of AI in one particularly creative human activity—the appreciation and production of art. AI might not seem well suited for such aesthetic engagement; however, it would be premature to relegate AI to a minor role. In what follows, I survey what it means for humans to appreciate and produce art, what AI seems capable of, and how the two might converge.

## Manuscript

### Agency and purpose in art

If an average person in the US were asked to name an artistic genius they might mention Michelangelo or Picasso. Having accepted that they are geniuses, the merit of their work is given the benefit of the doubt. A person might be confused by a cubist painting, but might be willing to keep their initial confusion at bay by assuming that Picasso knew what he was doing Art historical narratives value individual agency (Fineberg, 1995). By agency, I mean the choices a person makes, their intentionality, motivations, and the quality of their work. Even though some abstract art might look like it could be made by children, viewers distinguish the two by making inferences about the artists’ intentionality (Hawley-Dolan and Winner, 2011).

Given the importance we give to the individual artist, it is not surprising that most people react negatively to forgeries (Newman and Bloom, 2012). This reaction, even when the object is perceptually indistinguishable from an original, underscores the importance of the original creator in conferring authenticity to art. Authenticity does not refer to the mechanical skills of a painter. Rather it refers to the original conception of the work in the mind of the artist. We value the artist’s imagination and their choices in how to express their ideas. We might appreciate the skill involved in producing a forgery, but ultimately devalue such works as a refined exercise in paint-by-numbers.

Children care about authenticity. They value an original object and are less fond of an identical object if they think it was made by a replicator (Hood and Bloom, 2008). Such observations suggest that the value of an original unique object made by a person rather than a machine is embedded in our developmental psychology. This sensibility persists among adults. Objects are typically imbued with something of the essence of its creator. People experience a connection between the creator and receiver transmitted through the object, which lends authenticity to the object (Newman et al., 2014; Newman, 2019).

The value of art made by a person rather than a machine also seems etched in our brains. People care about the effort, skill, and intention that underly actions (Kruger et al., 2004; Snapper et al., 2015); features that are more apparent in a human artist than they would be with a machine. In one study, people responded more favorably to identical abstract images if they thought the images were hanging in a gallery than if they were generated by a computer (Kirk et al., 2009). This response was accompanied by greater neural activity in reward areas of the brain, suggesting that the participants experienced more pleasure if they thought the image came from a gallery than if it was produced by a machine. We do not know if such responses that were reported in 2009, will be true in 2029 or 2059. Even now, biases against AI art are mitigated if people anthropomorphize the machine (Chamberlain et al., 2018). As AI art develops, we might be increasingly fascinated by the fact that people can create devices that themselves can create novel images.

Before the European Renaissance, agency was probably not important for how people thought about art (Shiner, 2001). The very notion of art probably did not resemble how we think of artworks when we walk into a museum or a gallery. Even if the agency of an artist did not much matter, purpose did. Religious art conveyed spiritual messages. Indigenous cultures used art in rituals. Forms of a gaunt Christ on the crucifix, sensual carvings at Khajuraho temples, and Kongo sculptures of human forms impaled with nails, served communal purposes. Dissanayake (2008) emphasized the deep roots of ritual in the evolution of art. Purpose in art does not have to be linked to agency. We admire cave paintings at Lascaux or Alta Mira but do not give much thought to specific artists who made them. We continue to speculate about the purpose of these images.

Art is sometimes framed as “art for art’s sake,” as if it has no purpose. According to Benjamin (1936/2018) this doctrine, *l’art pour l’art*, was a reaction to art’s secularization. The attenuation of communal ritualistic functions along with the ease of art’s reproduction brought on a crisis. “Pure” art denied any social function and reveled in its purity.

Some of functions of art shifted from a communal purpose to individual intent. The Sistine Chapel, while promoting a Christian narrative, was also a product of Michelangelo’s mind. Modern and contemporary art bewilder many because the message of the art is often opaque. One needs to be educated about the point of a urinal on a pedestal or a picture of soup cans to have a glimmer as to why anybody considers these objects as important works of art. In these examples, intent of the artist is foregrounded while communal purpose recedes and for most viewers is hard to decipher. Even though 20th Century art often represented social movements, we emphasize the individual as the author of their message. Guernica, and its antiwar message, is attributed to an individual, even when embedded in a social context. We might ask, what was Basquiat saying about identity? How did Kahlo convey pain and death? How did depression affect Rothko’s art?

Would AI art have a purpose? As I will recount later, AI at the very least could be a powerful tool for an artist, perhaps analogous to the way a sophisticated camera is a tool for a fine art photographer. In that case, a human artist still dictates the purpose of the art. For a person using AI art generating programs, their own cultural context, their education, and personal histories influence their choices and modifications the initial “drafts” of images produced by the generator. If AI develops sentience, then questions about the purpose of AI art and its cultural context, if such work is even produced, will come to the fore and challenge our engagement with such art.

### Reproduction and access

I mentioned the importance of authenticity in how a child reacts to reproductions and our distaste for forgeries. These observations point to a special status for original artwork. For Benjamin (1936/2018) the original had a unique presence in time and place. He regarded this presence as the artwork’s “aura.” The aura of art depreciates with reproduction.

Reproduction has been an issue in art for a long time. Wood cuts and lithographs (of course the printing press for literature) meant that art could be reproduced and many copies distributed. These copies made art more accessible. Photography and film, vastly increased reproductions of and access to art images.

Even before reproductions, paintings as portable objects within a frame, increased access to art. These objects could be moved to different locations, unlike frescoes or mosaics which had to be experienced *in situ* (setting aside the removal of artifacts from sites of origin to imperial collections). Paintings that could be transported in a frame already diminished their aura by being untethered to a specific location of origin.

Concerns about reproduction take on a different force in the digital realm. These concerns extend those raised by photographic reproduction. Analog photography retains the ghost of an original- in the form of a negative. Fine art photography often limits prints to a specific number to impart a semblance of originality and introduce scarcity to the physical artifact of a print. Digital photography has no negative. A RAW file might be close. Copies of the digital file, short of being corrupted, are indistinguishable from an original file, calling into question any uniqueness contained in that original. Perhaps non-fungible tokens could be used to establish an original unique identifier for such digital files.

If technology pushes art toward new horizons and commercial opportunities push advances in technology, then it is hard to ignore the likelihood that virtual reality (VR) and augmented reality (AR) will have an impact on our engagement with art. The ease of mass production and commercial imperatives to make more, also renders the notion of the aura of an individual object or specific location in VR nonsensical. AI art, by virtue of being digital, will lack uniqueness and not have the same aura as a specific object tied to a specific time and place. However, the images will be novel. Novelty, as I describe later, is an important feature of creativity.

### Artificial intelligence in our lives

As I mentioned at the outset of this essay, machine learning and AI will have a profound effect on almost every aspect of what we do and how we live. Intelligence in current forms of AI is not like human cognition. AI as implemented in deep learning algorithms are not taught rules to guide the processing of their inputs. Their learning takes different forms. They can be supervised, reinforced, or unsupervised. For supervised learning, they are fed massive amounts of labeled data as input and then given feedback about how well their outputs match the desired label. In this way networks are trained to maximize an “objective function,” which typically targets the correct answer. For example, a network might be trained to recognize “dog” and learn to identify dogs despite the fact that dogs vary widely in color, size, and bodily configurations. After being trained on many examples of images that have been labeled *a priori* as dog, the network identifies images of dogs it has never encountered before. The distinctions between supervised, reinforcement learning, and unsupervised learning are not important to the argument here. Reinforcement learning relies on many trial-and-error iterations and learns to succeed from the errors it makes, especially in the context of games. Unsupervised learning learns by identifying patterns in data and making predictions based on past patterns in that are not labeled.

Artificial intelligence improves with more data. With massive information increasingly available from web searches, commercial purchases, internet posts, texts, official records, all resting on enormous cloud computing platforms, the power of AI is growing and will continue to do so for the foreseeable future. The limits to AI are availability of data and of computational power.

Artificial intelligence does some tasks better than humans. It processes massive amounts of information, generates many simulations, and identifies patterns that would be impossible for humans to appreciate. For example, in biology, AI recently solved the complex problem of three-dimensional protein folding from a two-dimensional code (Callaway, 2022). The output of deep learning algorithms can seem magical (Rich, 2022). Given that they are produced by complex multidimensional equations, their results resist easy explanation.

Current forms of AI have limits. They do not possess common sense. They are not adept at analytical reasoning, extracting abstract concepts, understanding metaphors, experiencing emotions, or making inferences (Marcus and Davis, 2019). Given these limits, how could AI appreciate or produce art? If art communicates abstract and symbolic ideas or expresses nuanced emotions, then an intelligence that cannot abstract ideas or feel emotions would seem ill-equipped to appreciate or produce art. If we care about agency, short of developing sentience, AI has no agency. If we care about purpose, the purpose of an AI system is determined by its objective function. This objective, as of now, is put in place by human designers and the person making use of AI as a tool. If we care about uniqueness, the easy reproducibility of digital outputs depreciates any “aura” to which AI art might aspire.

Despite these reasons to be skeptical, it might be premature to dismiss a significant role of AI in art.

### Art appreciation and production

What happens when people appreciate art? Art, when most powerful, can transform a viewer, evoke deep emotions, and promote new understanding of the world and of themselves. Historically, scientists working in empirical aesthetics have asked participants in their studies whether they like a work of art, find it interesting, or beautiful (Chatterjee and Cardilo, 2021). The vast repository of images, on platforms like Instagram, Facebook, Flicker, and Pinterest, have images labeled with people’s preferences. These rich stores of data, growing every day, mean that AI programs can be trained to identify underlying patterns in images that people like.

Crowd-sourcing beauty or preference risks produce boring images. In the 1990s, Komar and Melamid (1999) conducted a pre-digital satirical project in crowd-sourcing art preferences. They hired polling companies to find out what paintings people in 11 countries wanted the most. For Americans, they found that 44% of Americans preferred blue; 49% preferred outdoor scenes featuring lakes, rivers, or oceans; more than 60% liked large paintings; 51% preferred wild, rather than domestic, animals; and 56% said they wanted historical figures featured in the painting. Based on this information, the painting most Americans want showed an idyllic landscape featuring a lake, two frolicking deer, a group of three clothed strollers, and George Washington standing upright in the foreground. For many critics, *The Most Wanted Paintings* were banal. They were the kind of anodyne images you might find in a motel. Is the Komar and Melamid experiment a cautionary tale for AI?

Artificial intelligence would not be polling people the way that Komar and Melamid did. With a large database of images, including paintings from various collections, the training phase would encompass an aggregate of many more images than collecting the opinions of a few hundred people. AI need not be confined to producing banal images reduced to a low common denominator. Labels for images in databases might end up being far richer than the simple “likes” on Instagram and other social media platforms. Imagine a nuanced taxonomy of words that describe different kinds of art and their potential impacts on viewers. At a small scale, such projects are underway (Menninghaus et al., 2019; Christensen et al., 2022; Fekete et al., 2022). These research programs go beyond asking people if they like an image, or find it beautiful or interesting. In one such project, we queried a philosopher, a psychologist, a theologian, and art historian and a neuroscientist for verbal labels that could describe a work of art and labels that would indicate potential impacts on how they thought or felt. Descriptions of art could include terms like “colorful” or “dynamic” or refer to the content of art such as portraits or landscapes or to specific art historical movements like Baroque or post-impressionist. Terms describing the impact of art certainly include basic terms such as “like” and “interest,” but also terms like “provoke,” or “challenge,” or “elevate,” or “disgust.” The motivation behind such projects is that powerful art evokes nuanced emotions beyond just liking or disliking the work. Art can be difficult and challenging, and such art might make some viewers feel anxious and others feel more curious. Researchers in empirical aesthetics are increasing focused on identifying a catalog of cognitive and emotional impacts of art. Over the next few years, a large database of art images labeled with a wide range of descriptors and impacts could serve as a training set for an art appreciating AI. Since such networks are adept at extracting patterns in vast amounts of data, one could imagine a trained network describing a novel image it is shown as “playing children in a sunny beach that evokes joy and is reminiscent of childhood summers.” The point is that AI need not know what it is looking at or experience emotions. All it needs to be able to do is label a novel image with descriptions and impacts- a more complex version of labeling an image as a brown dog even if it has never seen that particular dog before.

Can AI, in its current form, be creative? One view is that AI is and will continue to be good at automated but not creative tasks. As AI disrupts work and replaces jobs that involve routine procedures, the hope is that creative jobs will be spared. This hope is probably not warranted.

Sequence transduction or transformer models are making strides in processing natural language. Self-GPT-3 (generative pre-trained transformers) as of now building on 45 terabytes of data can produce text based on the likelihood of words co-occurring in sequence. The words produced by transformer models can seem indistinguishable from sentences produced by humans. GPT-3 transformers can produce poetry, philosophical musings, and even self-critical essays (Thunström, 2022).

The ability to use text to display images is the first step in producing artistic images. DALL-E 2, Imagen, Midjourney, and DreamStudio are gaining popularity as art generators that make images when fed words (Kim, 2022). To give readers, who might not be familiar with the range of AI art images, a sense of these pictures I offer some examples.

**FIGURE 1:**
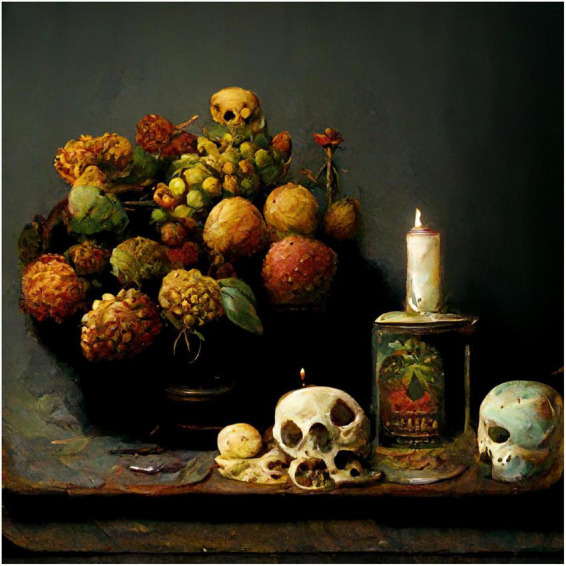
Midjourney image generated to the prompt “a still life with fruit, flowers, a vase, dead game, a candle, and a skull in a Renaissance style”.

The first set of images were made using Midjourney. I started with the prompt “a still life with fruit, flowers, a vase, dead game, a candle, and a skull in a Renaissance style” (Figure 1). The program generates four options, from which I picked the one that came closest to how I imagined the image. I then generated another four variations from the one I picked and chose the one I liked best. The upscaled version of the figure is included.

**FIGURE 2:**
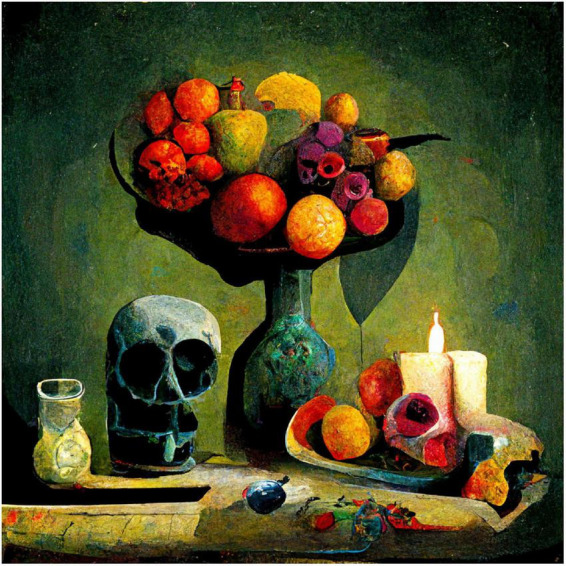
Midjourney image generated to the prompt “a still life with fruit, flowers, a vase, dead game, a candle, and a skull in an Expressionist style”.

**FIGURE 3:**
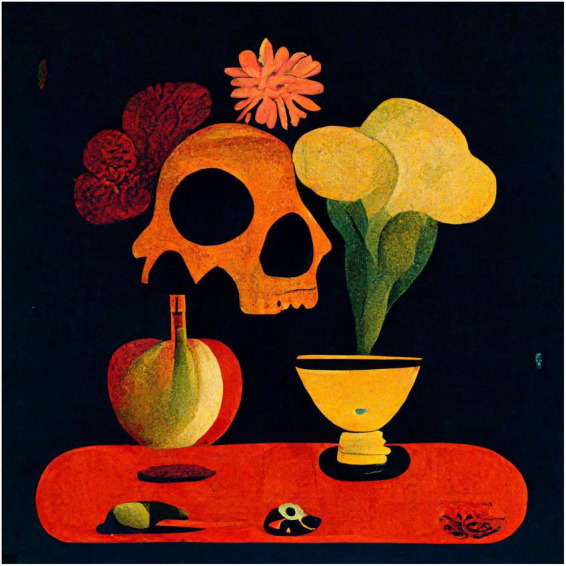
Midjourney image generated to the prompt “a still life with fruit, flowers, a vase, dead game, a candle, and a skull in a Pop-art style”.

**FIGURE 4:**
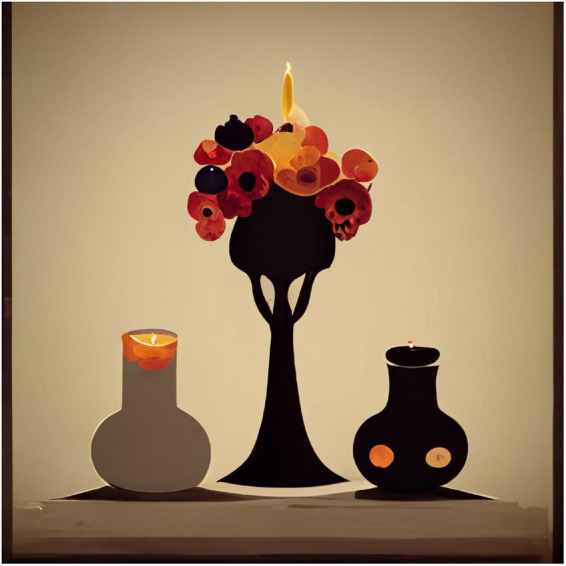
Midjourney image generated to the prompt “a still life with fruit, flowers, a vase, dead game, a candle, and a skull in a Minimalist style”.

To show variations of the kind of images produced, I used the same procedures and prompts, except changing the style to Expressionist, Pop-art, and Minimalist (Figures 2–4).

“To show how one might build up an image I used Open AI’s program Dall-E, to generate an image to the prompt, “a Surreal Impressionist Landscape.” Then using the same program, I used the prompt, “a Surreal Impressionist Landscape that evokes the feeling of awe.” To demonstrate how different programs can produce different images to the same prompt,” a Surreal Impressionist Landscape that evokes the feeling of awe” I include images produced by Dream Studio and by Midjourney.

**FIGURE 5:**
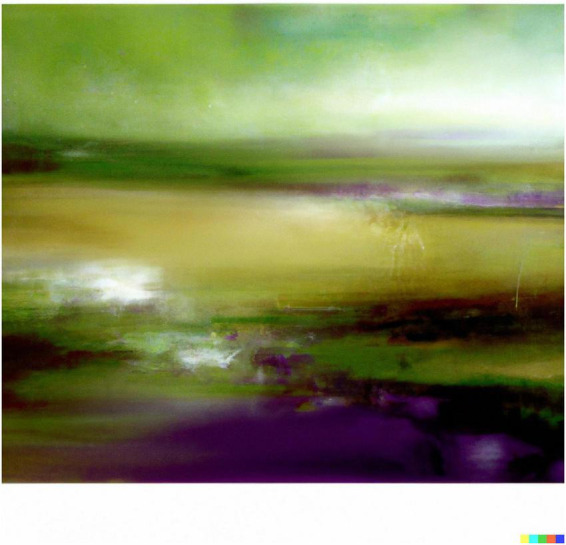
Dall-E generated image to the prompt “a Surreal Impressionist Landscape”.

**FIGURE 6:**
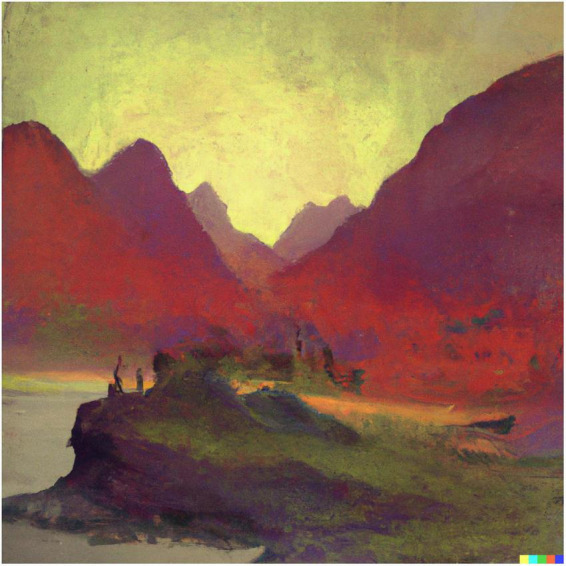
Dall-E generated image to the prompt “a Surreal Impressionist Landscape that evokes the feeling of awe”.

**FIGURE 7:**
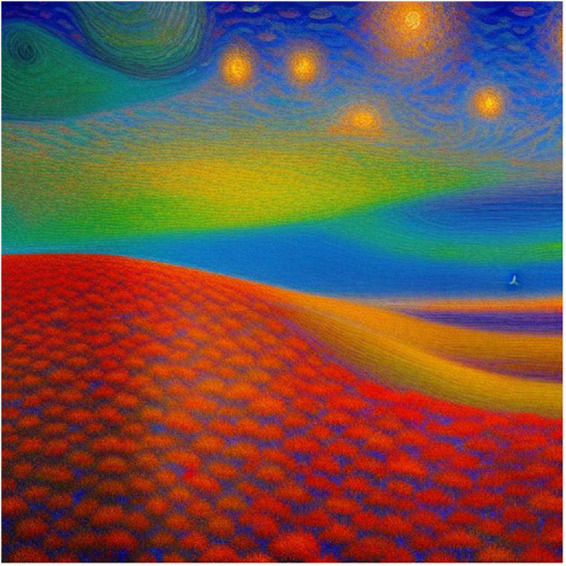
Dream Studio generated image to the prompt “a Surreal Impressionist Landscape that evokes the feeling of awe”.

**FIGURE 8:**
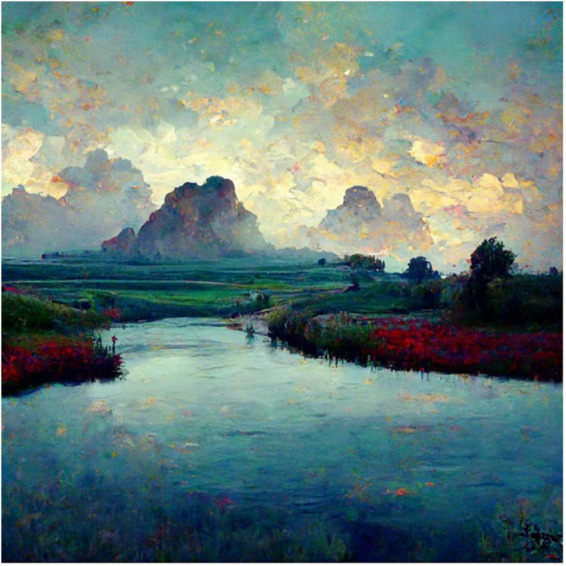
Midjourney generated image to the prompt “a Surreal Impressionist Landscape that evokes the feeling of awe”.

Regardless of the merits of each individual image, they only took a few minutes to make. Such images and many other produced easily could serve as drafts for an artist to consider the different ways they might wish to depict their ideas or give form to their intuitions (Figures 5–8). The idea that artists use technology to guide their art is not new. For example, Hockney (2001) described ways that Renaissance masters used technology of their time to create their work.

Unlike the imperative for an autonomous vehicle to avoid mistakes when it needs to recognize a child playing in the street, art makes no such demands. Rather, art is often intentionally ambiguous. Ambiguity can fuel an artworks’ power, forcing viewers to ponder what it might mean. What then will be the role of the human artist? Most theories of creative processing include divergent and convergent thinking (Cortes et al., 2019). Divergent thinking includes coming up with many possibilities. This phase can also be thought of as the generative or imaginative phase. A commonly used laboratory test is the Alternative Uses Test (Cortes et al., 2019). This test asks people to offer as many uses of a common object, like a brick, that they can imagine. The more uses, that a person can conjure up, especially when they are unusual, is taken as a measure of divergent thinking and creative potential. When confronting a problem that needs a creative solution, generating many possibilities doesn’t mean that they are the right or the best one. An evaluative phase is needed to narrow the possibilities, to converge on a solution, and to identify a useful path forward. In producing a work of art, artists presumably shift back and forth between divergent and convergent processes as they keep working toward their final work.

An artist could use text-to-image platforms as a tool (Kim, 2022). They could type in their intent and then evaluate the possible images generated, as I show in the figures. They might tweak their text several times. The examples of images included here using similar verbal prompts show how the text can be translated into images differently. Artists could choose which of the images generated they like and modify them. The divergent and generative parts of creative output could be powerfully enhanced by using AI, while the artist would evaluate these outputs. AI would be a powerful addition to their creative tool-kit.

Some art historians might object that art cannot be adequately appreciated outside its historical and cultural context. For example, Picasso and Matisse are better understood in relation to Cezanne. The American abstract expressionists are better understood as expressing an individualistic spirit while still addressed universal experiences; a movement to counter Soviet social realism and its collective ethos. We can begin to see how this important objection might be dealt with using AI. “Creative adversarial networks” can produce novel artworks by learning about historic art styles and then intentionally deviating from them (Elgammal et al., 2017). These adversarial networks would use other artistic styles as a contextual springboard from which to generate images.

Artificial intelligence and human artists might be partners (Mazzone and Elgammal, 2019), rather than one serving as a tool for the other. For example, in 2015 Mike Tyka created large-scale artworks using Iterative DeepDream and co-founded the Artists and Machine Intelligence program at Google. Using DeepDream and GANs he produced a series “Portraits of Imaginary People,” which was shown at ARS Electronica in Linz, Christie’s in New York and at the New Museum in Karuizawa (Japan) (Interalia Magazine, 2018). The painter Pindar van Arman teaches robots to paint and believes they augment his own creativity. Other artists are increasingly using VR as an enriched and immersive experience (Romano, 2022).

Kinsella (2018) Christie’s in New York sold an artwork called *Portrait of Edmond de Belamy* for $432,500. The portrait of an aristocratic man with blurry features was created by a GAN from a collective called Obvious. It was created using the WikiArt dataset that includes fifteen thousand portraits from the fourteenth to the twentieth century. Defining art has always been difficult. Art does not easily follow traditional defining criteria of having sufficient and necessary features to be regarded as a member of a specific category, and may not be a natural kind (Chatterjee, 2014). One prominent account of art is an institutional view of art (Dickie, 1969). If our social institutions agree that an object is art, then it is. Being auctioned and sold by Christie’s certainly qualifies as an institution claiming that AI art is in fact art.

In 2017, Turkish artist Refik Anadol, collaborating with Mike Tyka, created an installation using GANs called “Archive Dreaming.” This installation is an immersive experience with viewers standing in a cylindrical room. He used Istanbul’s SALT Galeta online library with 1.7 million images, all digitized into two terabytes of data. The holdings in this library relate to Turkey from the 19th Century to the present and include photographs, images, maps, and letters. Viewers stand in a cylindrical room and can gaze at changing displays on the walls. They can choose which documents to view, or the passively watch the display in an idle state. In the idle state, the archive “dreams.” Generators produce new images that resemble the original ones, but never actually existed—an alternate fictional historical archive of Turkey imagined by the machine (Pearson, 2022).

### Concerns, further future, and sentient artificial intelligence

Technology can be misused. One downside of deep learning is that biases embedded in training data sets can be reified. Systematic biases in the judicial system, in hiring practices, in procuring loans are written into AI “predictions” while giving the illusion of objectivity. The images produced by Dall-E so far perpetuate race and gender stereotypes (Taylor, 2022). People probably do not vary much if asked to identify a dog, but they certainly do in identifying great art. Male European masters might continue to be lauded over women or under-represented minority artists and others of whom we have not yet heard.

On the other hand, current gatekeepers of art, whether at high-end galleries, museums, and biennales, are already biased in who and what art they promote. Over time, art through AI might become more democratized. Museums and galleries across the world are digitizing their collections. The art market in the 21st Century extends beyond Europe and the United States. Important shows as part of art’s globalization occur beyond Venice, Basel, and Miami—to now include major gatherings in Sao Paulo, Dakar, Istanbul, Sharjah, Singapore, and Shanghai. Beyond high profile displays, small galleries are digitizing and advertising their holdings. As more images are incorporated into training databases, including art from Asia, Africa, and South America, and non-traditional art forms, such as street art or textile art, what people begin to regard as good or great art might become more encompassing and inclusive.

Could art become a popularity contest? As museums struggle to keep a public engaged, they might use AI to predict which kinds of art would draw in most viewers. Such a use of AI might narrow the range of art that are displayed. Similarly, some artists might choose to make art (in the traditional way), but shift their output to what AI predicts will sell. Over time, art could lose its innovation, its subversive nature, and its sheer variety. The nature of the artist might also change if the skills involved in making art change. An artist collaborating with AI might use machine learning outputs for the divergent phase of their creations and insert themselves along with additional AI assessments in the convergent evaluative phases of producing art.

The need for artistic services could diminish. Artists who work as illustrators for books, technical manuals, and other media such as advertisement, could be replaced by AI generating images. The loss of such paying jobs might make it harder for some artists to pursue their fine art dreams if they do not have a reliable source of income.

Many experts working in the field believe that AI will develop sentience. Exactly how is up for debate. Some believe that sentience can emerge from deep learning architectures given enough data and computational power. Others think that combining deep learning and classical programming, which includes the insertion of rules and symbols, is needed for sentience to emerge. Experts also vary in when they think sentience will emerge in computers. According to Ford (2021), some think it could be in a decade and others in over a 100 years. Nobody can anticipate the nature of that sentience. When Gary Kasparov (world Chess Champion at the time) lost to the program Deep Blue, he claimed that he felt an alien intelligence (Lincoln, 2018). Deep Blue was no sentient AI.

Artificial intelligence sentience will truly be an alien intelligence. We have no idea how or whether sentient AI will engage in art. If they do, we have no idea what would motivate them and what purpose their art would have. Any comments about these possibilities are pure speculation on my part.

Sentient AI could make art in the real world. Currently, robots find and move objects in large warehouses. Their movements are coarse and carried out in well-controlled areas. A robot like Rosey, the housekeeper in the Jetsons cartoon, is far more difficult to make since it has to move in an open world and react to unpredictable contingencies. Large movements are easier to program than fine movements, precision grips, and manual dexterity. The difficulty in making a robot artist would fall somewhere between a robot in an Amazon warehouse and Rosey. It would not have to contend with an unconstrained environment in its “studio.” It would learn to choose and grip different brushes and other instruments, manipulate paints, and apply them to a canvas that it stretched. Robot arms that draw portraits have been programed into machines (Arman, 2022). However, sentient AI with intent would decide what to paint and it would be able to assess whether its output matched its goal- using generative adversarial systems. The art appreciation and art production abilities could be self-contained within a closed loop without involving people.

Sentient AI might not bother with making art in the real world. Marc Zuckerberg would have us spend as much time as possible in a virtual metaverse. Sentient AI could create art residing in fantastical digital realms and not bother with messy materials and real-world implementation. Should sentient AI or sentient AIs choose to make art for whatever their purpose might be, humans might be irrelevant to the art making and appreciating or evaluating loop.

Ultimately, we do not know if sentient AI will be benevolent, malevolent, or apathetic when it comes to human concerns. We don’t know if sentient AI will care about art.

## Conclusion

As AI continues to insinuate itself in most parts of our lives, it will do so with art (Agüera y Arcas, 2017; Miller, 2019). The beginnings of art appreciation and production that we see now, and the examples provided in the figures, might be like the video game Pong that was popular when I was in high school. Pong is a far cry from the rich immersive quality of games like Minecraft in the same way that Dall-E and Midjourney images might be a far cry from a future art making and appreciating machine.

The idea that creative pursuits are an unassailable bastion of humanity is untenable. AI is already being used as a powerful tool and even as a partner for some artists. The ongoing development of aesthetically sensitive machines will challenge our views of beauty and creativity and perhaps our understanding of the nature of art.

## Author contributions

The author confirms being the sole contributor of this work and has approved it for publication.
